# Use of pumpkin seed (*Cucurbita pepo*) versus placebo and versus alpha-blockers in the management of benign prostatic hyperplasia: a systematic review and meta-analysis of randomized controlled trials

**DOI:** 10.1590/acb416626

**Published:** 2026-11-09

**Authors:** Bianca Caroline Benedicto, Beatriz Taveira Constantinou, Amauri Moreira Leite, Soraya Hussein Orra, Beatriz Sanches Molina, Tayná Pereira Leite de Almeida, Beatriz Brandão da Silva Navas, Rodrigo Afonso da Silva Sardenberg, Fernando Gonçalves de Almeida, Ronaldo Soares Maia, José Arnaldo Shiomi da Cruz

**Affiliations:** 1 Universidade Nove de Julho – School of Medicine – Urology Department – São Bernardo do Campo (SP) – Brazil.; 2 Hapvida NotreDame – Research Department – São Paulo (SP) – Brazil; 3 Universidade Federal de São Paulo – Escola Paulista de Medicina – Urology Department – São Paulo (SP) – Brazil.

**Keywords:** Cucurbita Pepo, Prostatic Hyperplasia, Lower Urinary Tract Symptoms, Phytotherapy, Adrenergic Alpha-Antagonists, Plant Extracts

## Abstract

**Purpose:**

To compare pumpkin seed (*Cucurbita pepo*) with placebo and, separately, with alpha-blockers in men with benign prostatic hyperplasia-related lower urinary tract symptoms.

**Methods:**

Five databases were searched to 30 April 2025 for randomized controlled trials (RCTs). The primary outcome was change in international prostate symptom score (IPSS), and secondary outcomes included maximum urinary flow (Qmax), prostate volume, prostate-specific antigen (PSA) and adverse events. Each comparator was analyzed separately by random-effects meta-analysis, with RoB 2 for risk of bias.

**Results:**

Eight RCTs (2,170 men) were included. *Versus* placebo, pumpkin seed reduced IPSS at three months (mean difference -0.62, 95% confidence interval [95%CI] -1.15 to -0.09) but not at six months; *versus* alpha-blockers there was no difference (1.91, IC95% -0.24–4.07). Qmax improved *versus* placebo only; no other outcome differed from alpha-blockers. Adverse events were fewer with pumpkin seed in one head-to-head trial (odds ratio = 0.21, IC95% 0.07–0.63).

**Conclusion:**

Pumpkin seed offers at most a small sub-clinical IPSS benefit over placebo, not maintained at six months, and no significant difference from alpha-blockers. Certainty was low to very low; equivalence is not established.

## Introduction

Benign prostatic hyperplasia (BPH) is a histopathological condition characterized by non-malignant growth of the prostate gland in its transition zone, resulting from proliferation of both epithelial and stromal cells^1^. It is the most frequent benign lower urinary tract condition in men, and its prevalence rises steeply with age, affecting up to 90% of men by 85 years old^2^.

When prostatic enlargement obstructs the prostatic urethra, patients develop lower urinary tract symptoms (LUTS)—weak stream, hesitancy, urgency, nocturia—, which substantially impair quality of life^3-5^. The American Urological Association and the European Association of Urology recommend watchful waiting and lifestyle modification first, followed by pharmacological treatment with alpha-blockers; surgery is reserved for inadequate response or complications. Although effective, alpha-blockers and 5α-reductase inhibitors may cause hypotension, dizziness, fatigue, and sexual adverse effects, compromising quality of life and adherence^6,7^.

Interest in phytotherapy for BPH has grown with fewer adverse effects. Pumpkin seed (*Cucurbita pepo*, Cucurbitaceae) is rich in phytosterols proposed to inhibit 5α-reductase, reducing conversion of testosterone to dihydrotestosterone and, plausibly, the hyperplastic drive on prostatic tissue^8,9^. Since previous reviews have combined heterogeneous comparators and formulations, the real clinical effect remains uncertain. We therefore compared pumpkin seed oil/extract with placebo and, as a separate question, with alpha-blockers in men with BPH.

The objective of this study was to determine, in two independent random-effects meta-analyses, whether pumpkin seed (*C. pepo*) oil or extract improves international prostate symptom score (IPSS) and secondary urological outcomes *versus placebo*, and separately, whether it is comparable to alpha-blockers, in men with BPH/LUTS.

## Methods

### Protocol and reporting

This systematic review and meta-analysis were conducted according to the Cochrane Handbook and reported per Preferred Reporting Items for Systematic reviews and Meta-Analyses (PRISMA) 2020. The completed PRISMA 2020 checklist and full search strategies together with the extracted dataset and the analysis code, is deposited in the Zenodo repository^10^. The protocol was registered in PROSPERO (CRD420261445031; https://www.crd.york.ac.uk/prospero/display_record.php?RecordID=1445031).

### Ethics approval

As the study used only published, aggregate data, institutional ethics approval and informed consent were not required.

### Search strategy

We searched PubMed, Embase, Scopus, the Cochrane Library, and Google Scholar from inception to 30 April 2025 using the strategy: (“pumpkin seed oil” OR “pumpkin seed extract” OR “*Cucurbita pepo*” OR “pumpkin seeds”) AND (“benign prostatic hyperplasia” OR BPH OR “lower urinary tract symptoms” OR LUTS). Database-specific adaptations of this strategy are reported as supplementary material^10^. Reference lists of included studies and prior reviews were hand-searched; no additional eligible records were identified.

Full reports were sought for every record that was not excluded at title and abstract screening. Part of the yield from the Cochrane Library (CENTRAL) and Google Scholar consists of records for which no full report exists—trial-registry entries, conference abstracts, theses, book chapters and product monographs—, and these were counted as reports not retrieved in the flow diagram. The composition of the non-retrieved set and a formal assessment of its potential impact on the findings are reported as supplementary material^10^.

### Eligibility criteria

The criteria for inclusion were:

- Randomized controlled trials (RCTs);
- Comparing pumpkin seed oil/extract with placebo and/or with alpha-blockers;
- Adult men with BPH;
- Reporting ≥ 1 outcome of interest (IPSS, urinary flow, post-void residual volume (PVR), prostate volume, quality of life, adverse events).

The criteria for exclusion were:

- Animal or *in-vitro* studies;
- Reviews, meta-analyses, case reports, letters;
- No comparator group;
- Non-randomized study designs.

Studies in which pumpkin seed was administered as part of a multi-herb preparation were eligible but were identified as such and interpreted with this caveat (see the section “Limitations”).

### Comparisons and endpoints

Two comparisons were analyzed separately:

- Pumpkin seed *versus* placebo;
- Pumpkin seed *versus* alpha-blocker.

All comparison-B trials were head-to-head (pumpkin seed replacing the alpha-blocker rather than being added to it). The primary endpoint was change in IPSS at three and six months; secondary endpoints were Qmax, PVR, prostate volume, prostate-specific antigen (PSA), quality of life, and adverse events. For the IPSS interpretation, a minimal clinically important difference (MCID) of approximately 3 points was used as the benchmark^11^.

### Screening and data extraction

After deduplication in EndNote Online (version 20), two independent reviewers (BCB and BTC) screened titles and abstracts, then full texts; disagreements were resolved by a third reviewer (JASC). Two reviewers (BCB and BTC) independently extracted data using a predefined form, with disagreements resolved by a third reviewer (JASC). Risk of bias was assessed independently by BCB and AMLJ, with disagreements resolved by consensus.

### Risk of bias and certainty of evidence

All included RCTs were assessed with the Cochrane RoB 2 tool. Leave-one-out and Baujat analyses were used to explore the influence of individual trials and potential instability of the pooled estimates. The certainty of evidence for each outcome and comparison was rated with the GRADE approach and summarized in a summary-of-findings table in supplementary material^10^.

### Statistical analysis

Continuous outcomes are reported as mean difference (MD) with 95% confidence intervals (95%CI); adverse events as *odds ratio* (OR) with 95%CI (Mantel–Haenszel). Random-effects models used the restricted maximum-likelihood estimator of τ^2^ throughout, including the IPSS three-month model. Because the placebo and alpha-blocker comparisons address different clinical questions, they were analyzed as two independent meta-analyses per outcome. Primary forest plots display only comparator-specific subgroup estimates; no overall pooled estimate across comparators is shown or was interpreted as a treatment effect. For the GRANU trial^12^, which had two active arms (extract and seed) sharing a single placebo group, the two active arms were combined into one group using the formula for combining groups given in Section 6.5.2.10 of the Cochrane Handbook for Systematic Reviews of Interventions to avoid double counting the shared control. Between-study heterogeneity was quantified with τ2 and prediction intervals in addition to I^2^. Leave-one-out analysis and Baujat plots explored influence and instability and are reported as supplementary material^10^.

For PSA, because one control standard deviation appeared anomalous, sensitivity analyses correcting that value and excluding the study were performed. Small-study-effect (funnel/Egger) testing was planned only when ≥ 10 studies contributed to a single comparison; as no comparison reached that threshold, and because such tests are unreliable under high heterogeneity, they were not performed. Analyses used R 4.5.2 with the *meta* and *metafor* packages; significance was two-sided at *p* < 0.05.

## Results

### Study selection and characteristics

The search retrieved 615 records from five databases (PubMed 65, Embase 197, Scopus 89, Cochrane Library 164, Google Scholar 100). After removal of 287 duplicates, 328 records were screened at title and abstract level, and 233 were excluded. Among the 95 reports sought for retrieval, 51 could not be retrieved; 44 full texts were assessed for eligibility, and 36 were excluded (15 reviews, five non-comparative studies, 15 different interventions, one non-randomized design), leaving eight RCTs^12-19^ included in the meta-analysis^10^. One study initially judged eligible, by Fusco et al.^20^, was excluded during data extraction because its intervention was a multi-ingredient product based on *Cucurbita maxima*—not *Cucurbita pepo*—combined with capsicum, polygonum, and vitamins, and therefore did not meet the pumpkin-seed intervention criterion^10^ . A second study, Krakhotkin et al.^21^, was excluded because it was a prospective non-randomized cohort rather than an RCT; as this review was restricted to RCTs, it was not retained for narrative description or risk-of-bias assessment^10^ (Fig. 1).

**Figure 1:**
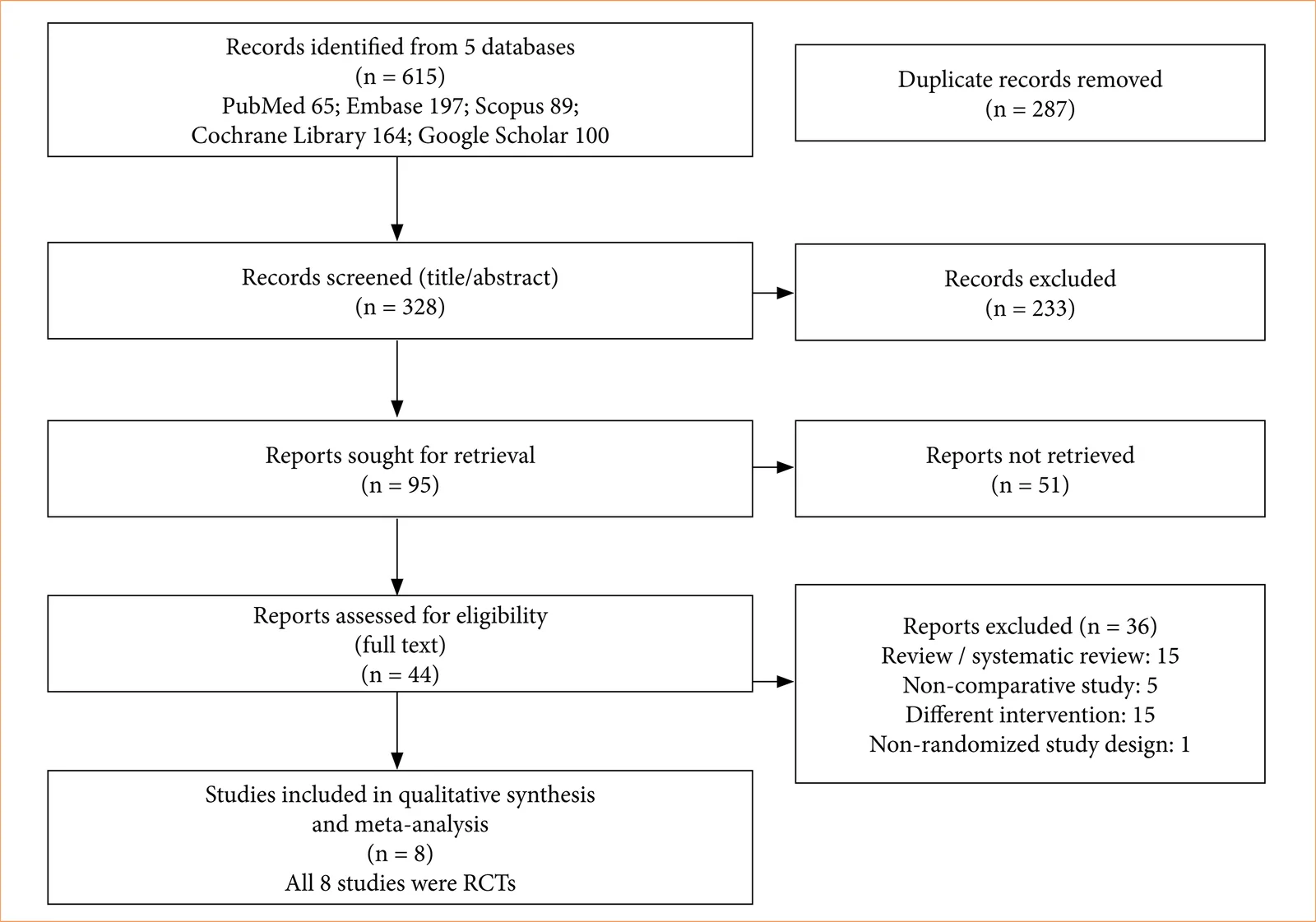
Preferred Reporting Items for Systematic reviews and Meta-Analyses (PRISMA) 2020 flow diagram of study selection.

RCT: randomized controlled trial. Source: Elaborated by the authors.

In total, 1,322 men received pumpkin seed oil/extract and 848 received placebo or an alpha-blocker; mean age was 62 years old. Baseline characteristics are in Table 1, and prostate volume and PSA in Table 2. Five studies used a placebo comparator^12,13,16,17,19^ and three an alpha-blocker, all three head-to-head^14,15,18^. A post hoc assessment of the non-retrieved records—comprising an analysis of the source composition of the search yield, a verification search re-run in PubMed on 11 September 2026, and a cross-check against prior syntheses of this literature—identified no additional randomized trial meeting the eligibility criteria^10^.

**Table 1:** Baseline characteristics of the included studies of pumpkin seed oil and extract *versus* placebo and/or alpha-blockers.

Authors	Country	Study design	Patients PS	Patients PLA or AB	Age PS (years)	Age PLA or AB (years)	Dose PS	Dose PLA or AB
Vahlensieck et al.^12^	Germany	RCT	848	417	65	66	500 mg (Prosta Fink)	1 capsule/day (placebo)
Hong et al.^13^	South Korea	RCT	16	7	48	53	320 mg/day	320 mg/day (placebo)
Shirvan et al.^14^	Iran	RCT	47	45	64	63	2 capsules (Prostafit)	2 capsules/day (prazosin)
Zerafatjou et al.^15^	Iran	RCT	39	34	64	62	720 mg/day	0.4 mg/day (tamsulosin)
Bach^16^	Germany	RCT	233	243	—	—	500 mg/day	1 capsule/day (placebo)
Carbin et al.^17^	Sweden	RCT	26	27	62	61	80 mg/day	2 capsules/day (placebo)
Machado-Leiva et al.^18^	Cuba	RCT	81	50	67	66	140 mg/day (Calprost)	2 mg/day (terazosin)
Coulson et al.^19^	Australia	RCT	32	25	63	64	160 mg	160 mg (placebo)

PS: pumpkin seed oil/extract; PLA: placebo; AB: alpha-blocker; RCT: randomized controlled trial.

Source: Elaborated by the authors.

**Table 2:** Prostate volume and PSA outcomes in included studies.

Authors	Prostate volume PS (mL)	Prostate volume PLA or AB (mL)	PSA PS (ng/mL)	PSA PLA or AB (ng/mL)
Vahlensieck et al.^12^	28.8	28.0	1.75	1.9
Hong et al.^13^	22.4	23.2	0.8	1.0
Shirvan et al.^14^	22.1	23.2	1.76	1.80
Zerafatjou et al.^15^	53.53	50.9	2.91	2.39
Bach^16^	34.8	35.2	2.6	2.6
Carbin et al.^17^	—	—	6.7	6.6
Machado-Leiva et al.^18^	38	57	11	14
Coulson et al.^19^	28.6	28.0	2.8	3.3

PS: pumpkin seed oil/extract; PLA: placebo; AB: alpha-blocker; PSA: serum prostate-specific antigen.

Source: Elaborated by the authors.

### Primary outcome: international prostate symptom score

*Versus* placebo, pumpkin seed produced a small but statistically significant IPSS reduction at three months (MD = -0.62 points, 95%CI -1.15 to -0.09; I^2^ = 0%, two studies; 95% prediction interval -4.07 to 2.82)—a difference well below the ~3-point MCID and therefore of limited clinical relevance—and no significant difference at six months (MD = 0.16 points, 95%CI -1.98–2.31; I^2^ = 84.3%; 95% prediction interval -22.74–23.07) (Fig. 2).

**Figure 2:**
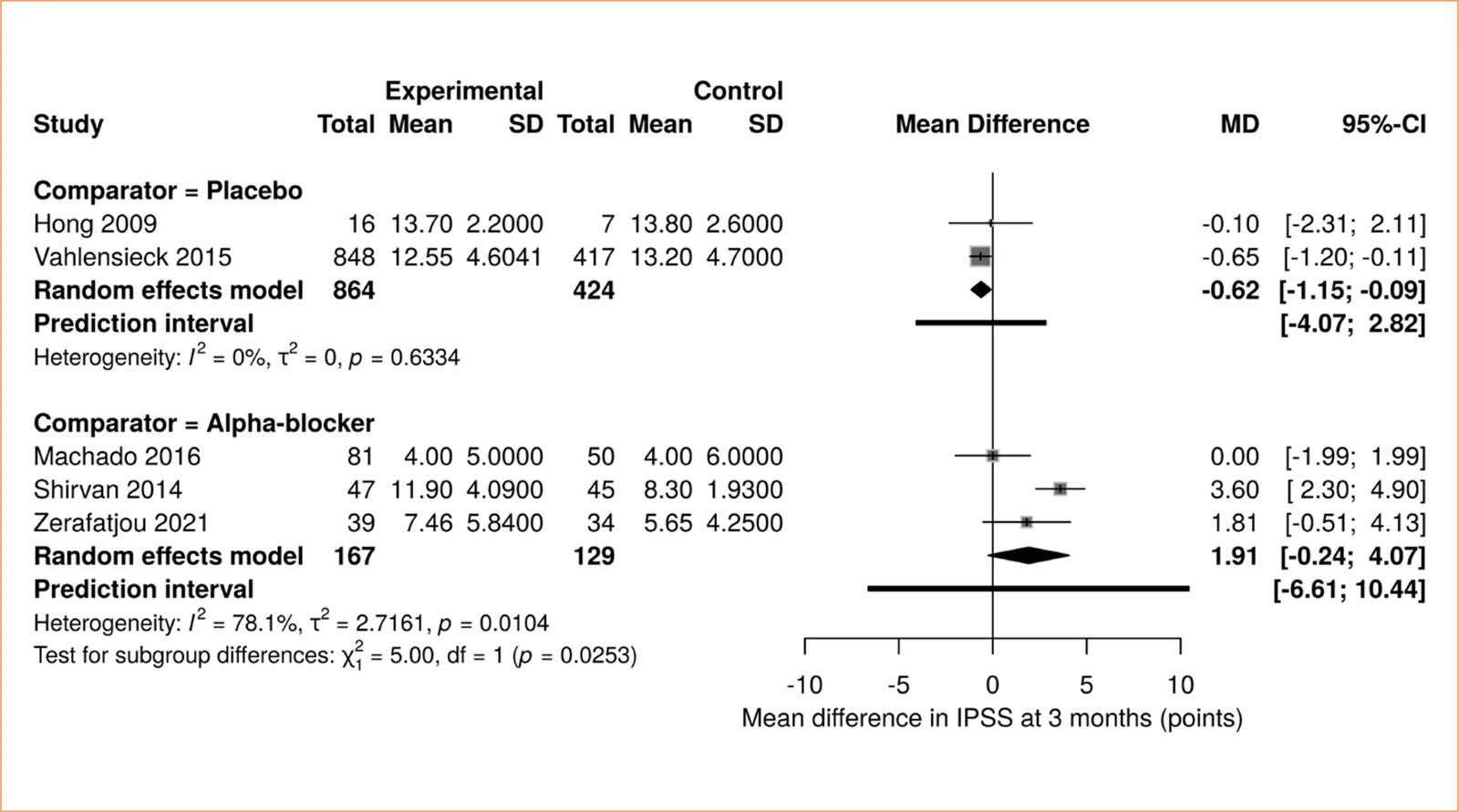
Forest plot of IPSS at three months, placebo and alpha-blocker comparisons shown separately.

SD: standard deviation; IPSS: international prostate symptom score; MD: mean difference; 95%CI: 95% confidence interval. Source: Elaborated by the authors.

*Versus* alpha-blockers, there was no significant difference at three months (MD = 1.91 points, 95%CI -0.24–4.07; I^2^ = 78.1%, three studies; 95% prediction interval -6.61 to 10.44; subgroup difference *versus* placebo *p* = 0.025) (Fig. 3). At six months, only one alpha-blocker trial remained^14^ (MD = 1.16 point, 95%CI -0.18–2.50), precluding a pooled estimate or heterogeneity assessment for that time point. Because the placebo subgroups pool only two studies, their prediction intervals are themselves imprecise (based on a t-distribution with as few as 1–2 degrees of freedom) and are reported to show the plausible range of true effects rather than as a precise bound; per current guidance, they should be read as reinforcing, not replacing, the visual heterogeneity pattern in the forest plots^22^. Leave-one-out and Baujat diagnostics for each comparison are shown in supplementary material^10^.

**Figure 3:**
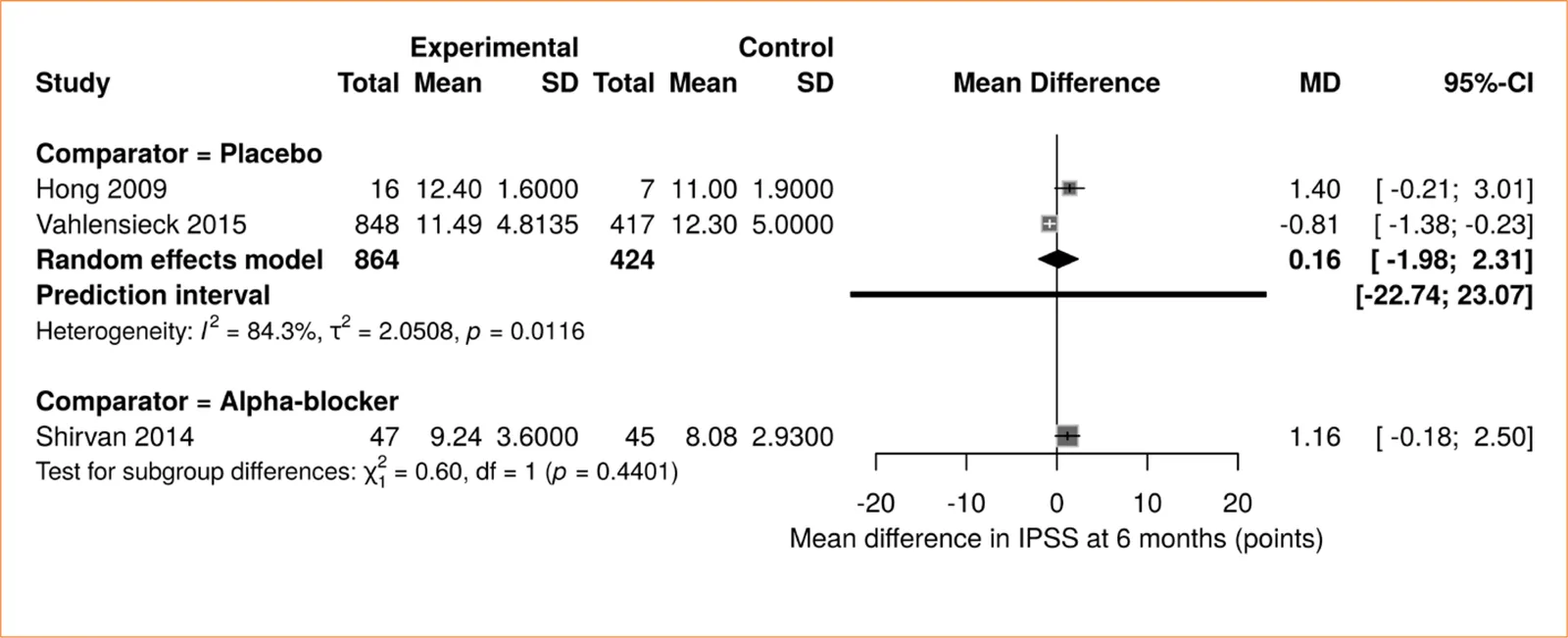
Forest plot of IPSS at six months, placebo and alpha-blocker comparisons shown separately.

SD: standard deviation; IPSS: international prostate symptom score; MD: mean difference; 95%CI: 95% confidence interval. Source: Elaborated by the authors.

### Maximum urinary flow

Qmax improved with pumpkin seed *versus* placebo (MD = 2.80 mL/s, 95%CI 1.26–4.34; single trial) but not *versus* alpha-blockers (MD = -3.34 mL/s, 95%CI -7.24–0.56; I^2^ = 85.6%; 95% prediction interval -45.19–38.50, based on only two studies and therefore highly imprecise), with a significant subgroup difference (*p* = 0.004) (Fig. 4). Leave-one-out and Baujat diagnostics are shown in supplementary material^10^.

**Figure 4:**
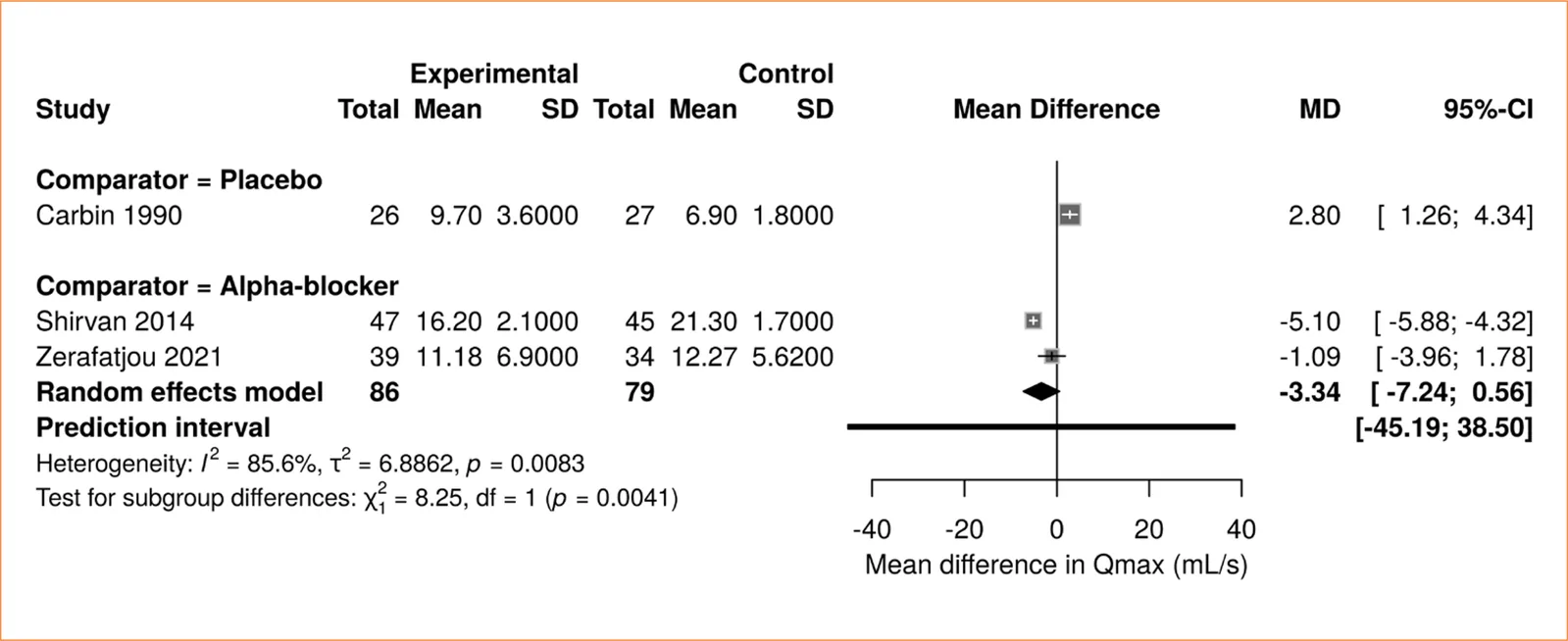
Forest plot of maximum urinary flow (Qmax), placebo and alpha-blocker comparisons shown separately.

SD: standard deviation; IPSS: international prostate symptom score; MD: mean difference; 95%CI: 95% confidence interval. Source: Elaborated by the authors.

### Secondary outcomes

Prostate volume (three alpha-blocker–comparator studies only; no placebo-controlled data) showed no significant difference^10^ (MD = -6.24 mL, 95%CI -23.16–10.68; I^2^ = 87.6%). PSA showed no significant difference *versus* alpha-blockers (MD = 0.38 ng/mL, 95%CI -0.78–1.53; I^2^ = 0%, two studies); this estimate was sensitive to the implausible control standard deviation reported by Shirvan et al.^14^ (see section “Limitations”)—correcting it to 0.40 shifted the alpha-blocker estimate to MD = -0.00 ng/mL (95%CI -0.18–0.18), while excluding the study outright left it materially unchanged (MD = 0.38 ng/mL, 95%CI -0.78–1.54), reflecting that this trial receives negligible weight (≈1%) either way^10^.

PVR (two alpha-blocker–comparator studies) showed no significant difference versus alpha-blockers (MD = -13.67 mL, 95%CI -29.04–1.70; I^2^ = 0%); a reduction was seen in the single placebo-controlled trial (MD = -27.50 mL, 95%CI -50.75 to -4.25), without a significant subgroup difference^10^ (*p* = 0.331). Quality of life (two alpha-blocker–comparator studies) showed no significant difference *versus* placebo (MD = 0.30 point, 95%CI -0.06–0.66; single study) or versus alpha-blockers^10^ (MD = 1.21 point, 95%CI -0.60–3.01; I^2^ = 96.1%).

### Adverse events

Two studies reported adverse-event counts (pumpkin seed versus comparator: Machado-Leiva et al.^18^ 5/81 *versus* 12/50; Bach^16^ 5/233 *versus* 0/243). *Versus* alpha-blockers, adverse events were significantly fewer with pumpkin seed in this single head-to-head trial (OR = 0.21, 95%CI 0.07–0.63); as this rests on one small trial, it should be regarded as preliminary and requires confirmation. The single placebo-controlled trial^16^ had a zero-event cell in the control arm (OR = 11.72, 95%CI 0.64–213.18) and should not be over-interpreted. Overall, reported adverse events were infrequent and mild, with no relevant cardiovascular or sexual events attributable to pumpkin seed.

### Risk of bias

Among the eight included RCTs (RoB 2; Fig. 5), two were low risk, three raised some concerns, and three were high risk.

**Figure 5:**
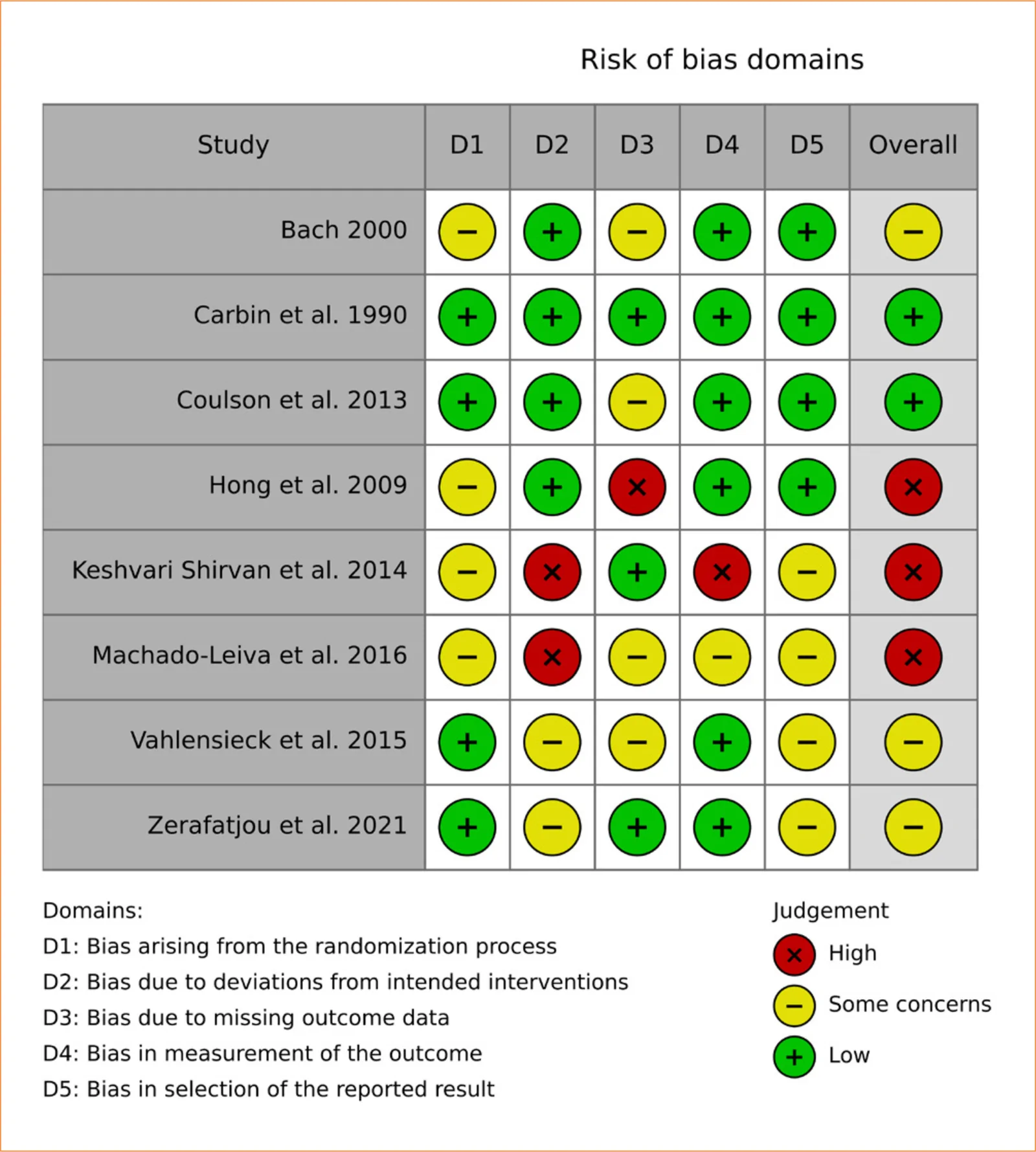
Risk of bias assessment of the included randomized controlled trials (RoB 2).

Source: Elaborated by the authors.

## Discussion

In this systematic review of eight RCTs (2,170 men), pumpkin seed (*C. pepo*) produced only a small, sub-clinical reduction in IPSS *versus* placebo at three months (MD = -0.62 point, below the ~3-point MCID), which was no longer detectable at six months, and no statistically significant difference versus alpha-blockers, although the subgroup contrast with placebo was itself significant (*p* = 0.025), consistent with a small, non-equivalent advantage of alpha-blockers over pumpkin seed on this outcome rather than proof that the two are interchangeable. A Qmax advantage over placebo was seen in a single trial but not against alpha-blockers. For prostate volume, PVR, and quality of life, no clinically relevant differences emerged when each comparator was examined separately; the PSA estimate versus alpha-blockers was likewise non-significant but rested on only two small trials and was materially affected by an implausible control standard deviation in one of them (see section “Limitations”).

These results are consistent with a systematic review and meta-analysis that likewise found no clinical or functional benefit of pumpkin-seed phytomedicines for BPH^23^, but temper the more optimistic tone of earlier narrative reviews of *C. pepo* ^24^, and are best explained by very high between-study heterogeneity together with differences in comparator, formulation, and design.

A central methodological point is that placebo and alpha-blocker comparisons answer different clinical questions and must not be collapsed into one treatment-effect estimate: a small benefit over placebo and a null or slightly unfavorable contrast against alpha-blockers cancel if forced into a single pool, producing an artefactually null overall figure. We therefore report and interpret the two comparisons independently throughout. Heterogeneity reached I^2^ = 78–96% for most alpha-blocker analyses with more than one contributing trial, so point estimates were interpreted alongside prediction intervals and the visual forest-plot pattern rather than in isolation, in line with current methodological guidance; several alpha-blocker subgroups rested on only one or two trials, so heterogeneity statistics and prediction intervals could not be computed or were correspondingly unstable.

The comparison against alpha-blockers warrants restraint despite all three contributing trials being true head-to-head comparisons (pumpkin seed replacing rather than being added to the alpha-blocker). With only two or three small trials per outcome, the absence of a significant difference must therefore not be read as equivalence or non-inferiority—no included trial was designed as a non-inferiority study, and confidence intervals remained wide enough to be compatible with a clinically relevant advantage for either arm.

A single head-to-head trial^18^ found significantly fewer adverse events with pumpkin seed than with an alpha-blocker (OR = 0.21, 95%CI 0.07–0.63), a signal consistent with the proposed mechanism (phytosterol-mediated 5α-reductase inhibition and anti-inflammatory effects)^25-28^. However, this rests on a single small trial, and the only placebo-controlled safety comparison^16^ had a zero-event control arm that precludes meaningful estimation. Safety conclusions therefore remain preliminary and should be confirmed in adequately powered trials rather than treated as an established advantage.

### Limitations

The dominant limitation is very high heterogeneity, where more than one trial contributed to an alpha-blocker subgroup, reflecting clinical and methodological diversity (baseline characteristics, formulations and doses, treatment duration, outcome definitions); the resulting prediction intervals are themselves wide, and for subgroups with only one or two studies they could not be computed or were imprecisely estimated. Most outcomes rested on one, two, or three studies per subgroup, producing unstable estimates that should be regarded as hypothesis-generating.

This review was restricted to RCTs: one study that met all other eligibility criteria^21^, a prospective non-randomized cohort that would have been rated at critical risk of bias on ROBINS-I, was excluded at the eligibility stage for this reason and is listed among excluded reports^10^ rather than contributing to any pooled estimate; readers consulting non-RCT-inclusive syntheses of this literature should expect somewhat different pooled figures for the alpha-blocker comparison as a result.

A second study initially considered for inclusion^20^ was excluded during data extraction because its intervention combined tamsulosin with a multi-ingredient product based on *Cucurbita maxima* rather than *Cucurbita pepo*. Three of the eight included RCTs were at high risk of bias on RoB 2. The control-group PSA standard deviation reported by Shirvan et al.^14^ (± 40 ng/mL for a mean of 1.78) is implausibly large relative to that study’s own baseline (± 0.90) and six-month (± 0.88) values and is reproduced here as published; because of its large variance the study received negligible weight (≈1%) in the pooled PSA estimate—correcting it to the plausible value of ± 0.40 shifted the alpha-blocker PSA estimate from MD = 0.38 to MD = -0.00 ng/mL, while excluding the study outright left the estimate materially unchanged (MD = 0.38 ng/mL), underscoring that this outcome is currently informed by very sparse data rather than a stable estimate. Isolated pumpkin seed products still varied in formulation and dose across trials, limiting comparability across the alpha-blocker studies even though all three were head-to-head.

Finally, among the 95 reports sought for retrieval, 51 (54%) could not be obtained, and we did not preserve an item-level record of which reports these were. Two features of the search make it likely that they consisted largely of material for which no full report exists: 264 of the 615 records (43%) came from the Cochrane Library (CENTRAL) and Google Scholar, which index trial-registry entries, conference abstracts, theses, book chapters and product monographs alongside journal articles, and the Google Scholar yield was capped at the first 100 relevant hits. Because that composition cannot be verified retrospectively, we assessed instead whether an eligible trial could have been lost^10^. A verification search re-run in PubMed on 11 September 2026—which also covers the 16 months elapsed since the search closing date—identified no randomized trial of *C. pepo* in BPH/LUTS that is not already included, and a cross-check against prior syntheses of this literature identified none either. Two of the eight included trials are not indexed in PubMed, one was published in German^16^ and one reporting a Cuban trial^18^ in a journal outside the major indexes, which argues against a systematic loss of non-English or non-indexed reports.

Finally, the three-month placebo comparison is dominated by a 1,265-participant multicenter trial, so unretrieved evidence would need precision comparable to the entire existing placebo evidence base together with an average IPSS advantage of about 5.5 points—roughly eight times the largest advantage among the contributing trials (0.65 points)—to move the pooled estimate to the ≈3-point MCID. Residual uncertainty remains and we do not claim to have excluded retrieval bias; we note only that, because published trials tend to show larger treatment effects than grey literature, any such bias would be expected to favor pumpkin seed rather than to have produced the conservative conclusions drawn here.

## Conclusion

Current evidence indicates that pumpkin seed (*C. pepo*) offers, at most, only a small sub-clinical symptomatic improvement over placebo (not maintained at six months) and no statistically significant difference from alpha-blockers on most outcomes in men with BPH/LUTS. A single small head-to-head trial suggested fewer adverse events with pumpkin seed than with an alpha-blocker, but this preliminary safety signal requires confirmation and should not be treated as an established advantage.

By GRADE, the certainty of evidence was low to very low for all outcomes owing to substantial heterogeneity where more than one trial contributed, risk of bias, and the small number of trials per comparison, and the data do not support claims of equivalence or non-inferiority to alpha-blockers. Adequately powered, standardized-formulation, non-inferiority RCTs are needed before pumpkin seed can be positioned in BPH treatment guidelines.

## Data Availability

The extracted dataset, the R analysis code and all supplementary material are openly available in the Zenodo repository at https://doi.org/10.5281/zenodo.23049447.
